# Molecular Design, Preparation, and Characterization of Fluoro-Containing Polyimide Ultrafine Fibrous Membranes with High Whiteness, High Thermal Stability, and Good Hydrophobicity

**DOI:** 10.3390/molecules27175447

**Published:** 2022-08-25

**Authors:** Zhen Pan, Han-li Wang, Hao-ran Qi, Yan-shuang Gao, Xiao-lei Wang, Xin-xin Zhi, Yan Zhang, Xi Ren, Jin-gang Liu

**Affiliations:** 1 School of Materials Science and Technology, China University of Geosciences, Beijing 100083, China; 2 Shandong Huaxia Shenzhou New Material Co., Ltd., Zibo 256401, China

**Keywords:** polyimide, ultrafine fibrous membranes, electrospinning, trifluoromethyl, whiteness index

## Abstract

Polymeric ultrafine fibrous membranes (UFMs) with high thermal stability and high whiteness are highly desired in modern optoelectronic applications. A series of fluoro-containing polyimide (FPI) UFMs with high whiteness, good thermal stability, and good hydrophobicity were prepared via a one-step electrospinning procedure from the organo-soluble FPI resins derived from a fluoro-containing dianhydride, 4,4′-(hexafluoroisopropylidene) diphthalic anhydride (6FDA), and various diamines containing either pendant trifluoromethyl (–CF_3_) groups or alicyclic units in the side chains. The obtained FPI UFMs, including FPI-1 from 6FDA and 3,5-diaminobenzotrifluoride (TFMDA), FPI-2 from 6FDA and 2′-trifluoromethyl-3,4′-oxydianiline (3FODA), FPI-3 from 6FDA and 1,4-bis[(4-amino-2-trifluoromethyl)phenoxy]benzene (6FAPB), FPI-4 from 4,4′-bis[(4-amino-2-trifluoromethyl)phenoxy]biphenyl (6FBAB), and FPI-5 from 6FDA and 4′-*tert*-butyl-cyclohexyl-3,5-diaminobenzoate (DABC) showed whiteness indices (WI) higher than 87.00 and optical reflectance values higher than 80% at the wavelength of 457 nm (R_457_), respectively. The FPI-5 UFM, especially, showed the highest WI of 92.88. Meanwhile, the prepared PI UFMs exhibited good hydrophobic features with water contact angles (WCA) higher than 105°. At last, the PI UFMs exhibited good thermal stability with glass transition temperatures (T_g_) higher than 255 °C, and the 5% weight-loss temperatures (T_5%_) higher than 510 °C in nitrogen.

## 1. Introduction

High-performance polymeric ultrafine fibers and the derived fabrics have been paid a wide range of attention in recent years as air or fluid filtration mediums in healthcare or environmental protection, gas or liquid separation membranes, functional membranes for energy devices, wound-dressing compounds in tissue engineering and therapy fields, and other functional components in high-tech areas [1,2,3,4,5,6]. Among various functional polymeric ultrafine fabrics, the species with high thermal resistance and high whiteness are brought into increasing focus in advanced optoelectronic applications [7,8,9,10]. Conventional polymeric ultrafine fibrous membranes (UFMs), including the electrospun poly(methyl methacrylate) (PMMA) [11], poly(ethylene terephthalate) (PET) [11], polyvinyl alcohol (PVA) [12], polylactic acid (PLA) [13], polycaprolactone (PCL) [14], polyvinyl chloride (PVC) [14], and polyurethane (PU) [15] membranes usually suffer from poor thermal stability, although they usually exhibit extremely high whiteness. Contrarily, standard high-temperature resistant polymeric UFMs, such as the electrospun poly(pyromellitic dianhydride-co-4,4′-oxydianiline) (PI_PMDA-ODA_) [16], polyphenylquinoxaline [17,18], and other heteroaromatic membranes, usually exhibit poor whiteness, although they usually possess excellent thermal stability at elevated temperatures. It is well established that the main factors affecting the whiteness of polymeric UFMs usually come from the physical and chemical features of the membranes. The former usually involves the fiber diameters, specific surface areas, fiber alignment, and other physical characteristics of the electrospun membranes. For example, electrospun UFMs usually exhibit much higher whiteness indices than those of polymeric films with the same chemical compositions, due to the diffuse reflection of the incident lights by the relatively randomly aligned fine fibers in UFMs [11]. The chemical factors usually originate from the intrinsic absorptions of the visible lights in polymers due to the intra- and inter-molecular charge transfer (CT) interaction in polymer chains [19,20]. The CT interactions are prominently featured in heteroaromatic polymers, such as the wholly aromatic polyimide (PI), in which strong CT interactions occur from the electron-donating diamine units to the electron-accepting dianhydride units [21]. The occurrence of CT interactions induces the light absorption, resulting in the high coloration and low whiteness of the PIs. On the other hand, for wholly aromatic PI UFMs, another important chemical factor is the effects of high-temperature cyclization from the poly(amic acid) (PAA) precursors to the final PIs [22]. An imidization temperature as high as 300 °C often deteriorates the optical properties of the derived PI UFMs [23]. Meanwhile, the dehydration reactions from PAA UFMs to PI UFMs often cause the adherence between the adjacent fibers, thus decreasing the scattered reflection of PI UFMs.

**Figure 1:**
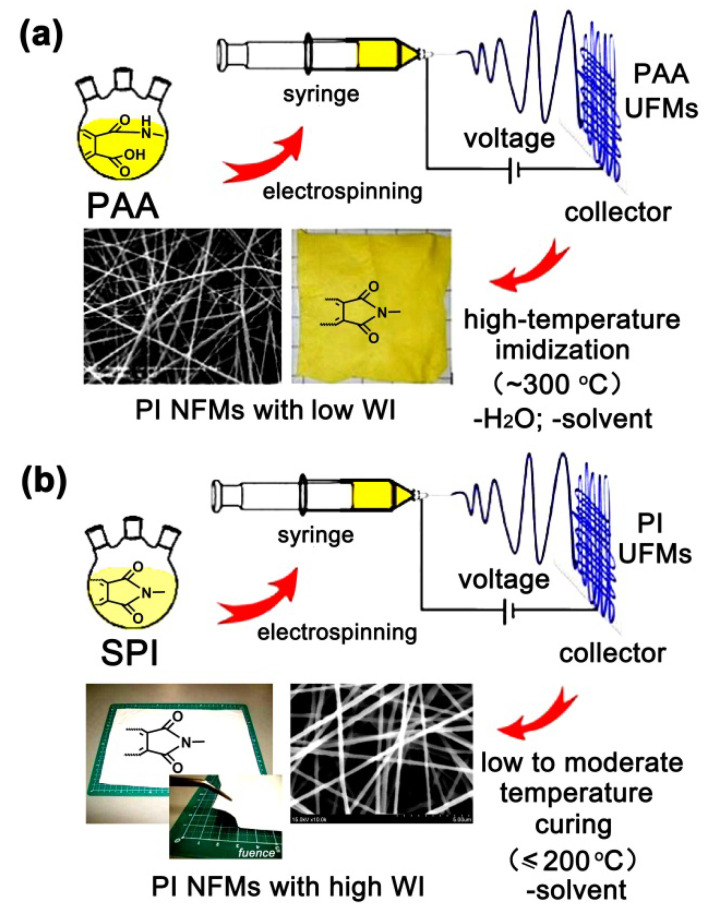
Effects of electrospun medium on the whiteness of PI UFMs. (**a**) PAA medium; (**b**) SPI medium.

In view of the origination of the poor whiteness of conventional electrospun PI UFMs, various modification works have been performed in the literature. Up until now, most of the works have focused on two pathways. The first is to prohibit the high-temperature oxidation or yellowing of the PI UFMs via developing preimidized PI resins as the starting materials for the fabrication of PI UFMs, as illustrated in Figure 1. Soluble PI (SPI) solutions are used as the electrospinning medium instead of the PAA precursors. Thus, the PI UFMs can be obtained by a one-step electrospinning procedure (Figure 1b). The post-treatment at temperatures below 200 °C is only to remove the residual solvent in the PI UFMs. By this alternative procedure, PI UFMs with improved high whiteness can often be obtained [24,25,26]. Secondly, in order to develop the PI UFMs with further enhanced whiteness, functional SPI resins were researched and developed. The intrinsic CT interactions in the PIs could be obviously reduced by the incorporation of structural units with high electronegativity (–CF_3_, etc.), low conjugation (alicyclic units, etc.), high molar volume (bulky substituents), or asymmetrical groups. Electrospun PI UFMs with excellent whiteness might be obtained by these types of functional PIs. In practical applications, the modification procedures mentioned above are often used together. Thus, high-whiteness PI NFMs with excellent combined properties could be obtained.

In the current work, as one of continuous efforts in developing high-performance electrospun PI UFMs for optoelectronic applications, a series of electrospun PI UFMs with high whiteness, good hydrophobic features, and good thermal stability were developed. For this target, fluoro-containing PI resins were first prepared from a fluoro-containing dianhydride, 6FDA and various diamines containing pendant trifluoromethyl (–CF_3_) groups, or alicyclic units in the side chains. Effects of the trifluoromethyl or alicyclic substituents on the solubility, thermal, and optical properties of the derived FPI UFMs were investigated in detail.

## 2. Results

### 2.1. Structural Characterization

**Figure 2:**
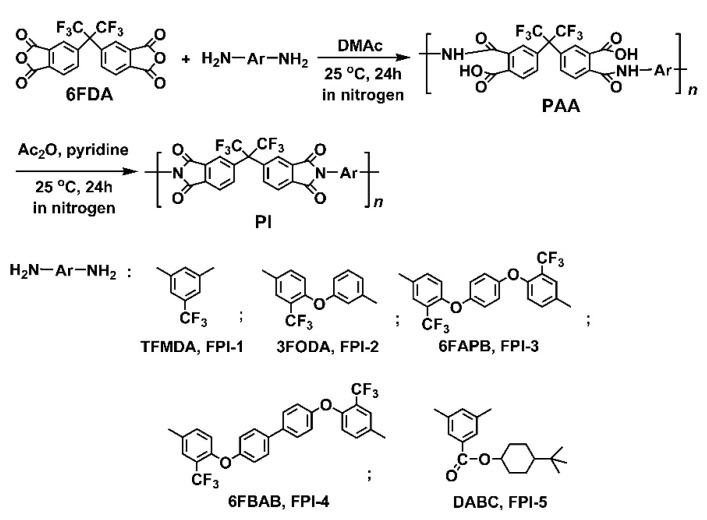
Preparation of fluoro-containing PI resins.

FPIs are well-known for their excellent combined properties, including good thermal and thermo-oxidative stability, high optical transparency of the films, low dielectric constants, good gas separation properties, and so on [27,28,29]. Thus, FPIs have been widely used as varnishes, films, adhesives, composites, aerogel, and other components in high-tech fields in the past decades [30]. However, research on the electrospun FPI UFMs has rarely been reported in the literature [31]. In recent years, with the ever-increasing requirements of high-performance UFMs for advanced optoelectronic fabrication in the energy and environmental fields, the research and development of functional FPI electrospun UFMs have received an emphatic focus. In the current research, five FPI UFMs were fabricated by electrospinning procedure. For this purpose, the corresponding organo-soluble FPI resins were synthesized first, with the route shown in Figure 2. 6FDA was polymerized with five diamines, respectively, to give the PAA precursors first. Then, PAAs were chemically dehydrated to afford the final SPI resins. All the polymerization proceeded homogeneously without the occurrence of gelling, precipitating, or other unstable phenomenon during the polycondensation, indicating the good solubility of PI resins in the reaction systems.

The obtained FPI resins showed intrinsic viscosities ([η]_inh_) higher than 0.80 dL/g (Table 1), revealing the high polymerization reactivity of the monomers. In addition, as shown in Table 1, FPI resins showed the number average molecular weights (M_n_) in the range of 5.37 × 10^4^–28.70 × 10^4^ g/mol and PDI values of 1.73–2.02. The moderate to high M_n_ values indicated that the aromatic diamines exhibited different reactivities during the polymerization with 6FDA under similar reaction conditions. FPI resins showed the decreasing M_n_ values with the order of FPI-5 > FPI-4 ≈ FPI-3 > FPI-2 > FPI-1. This trend is basically in good agreement with the reactivities of the diamines, which could be roughly estimated from the calculated highest occupied molecular orbital (HOMO) energy levels (ε_HOMO_) for the diamines, according to density functional theory (DFT)/B3LYP methods with Gaussian 09 software (Gaussian, Wallingford, CT, USA) using the 6-311G(d) basis set [32]. It has been well established that the ε_HOMO_ value of one diamine is closely related with the reactivity in the nucleophilic substitution reaction with the dianhydride. A higher ε_HOMO_ value for the diamine could roughly indicate higher reactivity of the diamine monomer [33]. According to the calculated ε_HOMO_ values of the diamines shown in Figure S1 (Supplementary Materials), the diamines showed a decreasing reactivity of DABC > 3FODA > 6FBAB > 6FAPB > TFMDA, which well explained the effects of different diamines on the molecular weights of FPI resins, except that of 3FODA. Although 3FODA showed high reactivity according to the simulation, FPI-2 derived from the diamine showed relatively lower M_n_ values. This might be due to the oxidation-sensitive nature of 3FODA diamine during storage in air environments. Oxidation of the amine groups might be accelerated by the *meta*-substituted molecular structure, resulting in slightly decreasing purity for the diamine.

All FPI resins were soluble in polar high-boiling-point solvents at room temperature, such as NMP, DMAc, and GBL. FPI-1–FPI-4 were also soluble in CPA with moderate boiling point. FPI-1 and FPI-2 were also soluble in chloroform with quite a low boiling point (Table 1). The good solubility of FPI resins in the solvents with different boiling points endowed them with good solution-processability within a wide temperature range, according to the practical applications. In the case of current research, electrospun FPI UFMs might be obtained at a relatively lower temperature compared to the conventional high-temperature imidization procedure of PAAs. The lower processing temperature of FPI UFMs could guarantee the whiteness to a great extent. At last, some of the good solvents for current FPI resins, such as CPA, showed low sensitivity to environmental humidity. This is quite beneficial for the practical electrospinning fabrication of FPI UFMs in humid environments. The good solubility of FPI resins is mainly attributed to the amorphous nature of the molecular chains, as could be deduced from the XRD spectra of the FPIs shown in Figure S2 (Smentary Materials). The incorporation of the bulky trifluoromethyl or *tert*-butyl-substituted cyclohexyl prohibited the ordered packing of the molecular chains of the polymers.

**Figure 3:**
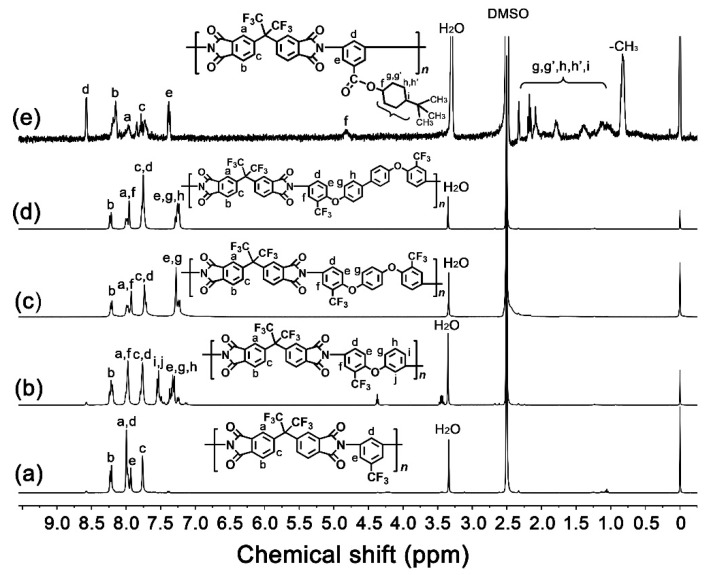
^1^H-NMR spectra of FPI resins. (**a**) FPI-1, (**b**) FPI-2, (**c**) FPI-3, (**d**) FPI-4, (**e**) FPI-5.

The chemical compositions of FPI resins were detected with ^1^H-NMR measurements, and the results are shown in Figure 3. All FPI resins were soluble in the NMR solvent of DMSO-*d*_6_ and revealed proton absorptions in the chemical shift range of 0.5–9.0 ppm. All the FPIs showed absorptions in the downfield regions in the spectra, except that of FPI-5, which showed clear signals in the upfield of 0.5–2.5 ppm due to the absortions of aliphatic or alicyclic protons in the side chains. The protons adjacent to the imide carbonyl groups and the trifluoromethyls (H_b_, H_a_ in FPI-1–FPI-4) showed the absorptions at the farther downfield regions in the spectra due to the electron-withdrawing nature of the groups. Contrarily, the protons adjacent to the ether linkages (H_e_, H_g_ in FPI-2–FPI-4) showed absorptions at the farther upfield areas in the spectra due to the electron-doanting nature of the groups. These structural characteristics were in good agreement with the expected structures for FPI resins.

**Figure 4:**
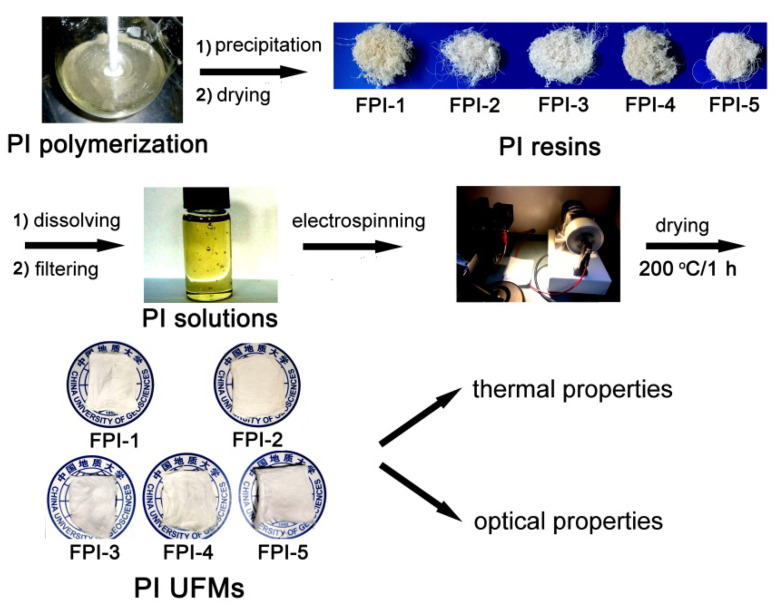
Fabrication procedure for the FPI UFMs.

**Figure 5:**
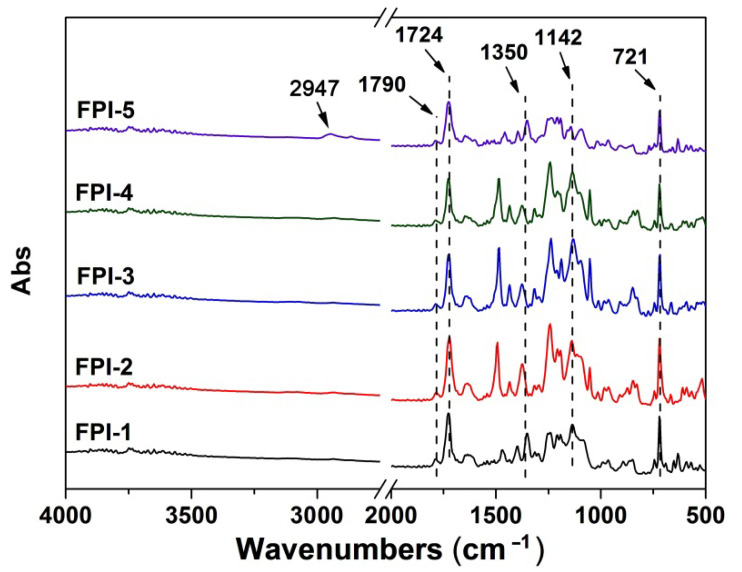
FTIR spectra of the FPI UFMs.

The developed FPI resins were dissolved into DMAc with the same solid content of 15 wt% to afford the FPI solutions for electrospinning fabrication, as shown in Figure 4. Flexible and tough FPI UFMs with high whiteness were all obtained according to the well-established electrospinning procedures, after being thermally dried at 200 °C for 1 h [24]. The chemical structures of FPI UFMs were confirmed by the FTIR measurements, as shown in Figure 5. The characteristic absorptions of imide rings, including those at 1790 cm^−1^ and 1724 cm^−1^, ascribed to the asymmetric and symmetric carbonyl stretching vibrations, respectively, as well as the ones at 1350 cm^−1^ assigned to the C–N stretching vibration, and the ones at 721 cm^−1^ assigned to the carbonyl bending vibration, were all clearly observed in the spectra. In addition, the characteristic absorptions of C–F bonds in trifluoromethyl or hexafluoroisopropyl units were also detected at 1142 cm^−1^. For FPI-5, the characteristic absorptions of the saturated C–H bonds in *tert*-butyl and cyclohexyl groups were detected at 2947 cm^−1^.

Figure S3 (Supplementary Materials) shows the micro-morphologies of fabricated FPI UFMs detected by SEM measurements, together with the statistical average fiber diameter (d_av_) of the UFMs shown in Table 2. It can be clearly observed that all FPI UFMs consisted of fine fibers with d_av_ values in the range of 360–1937 nm. The varieties of d_av_ values for PI UFMs were closely related with the M_n_ values of FPI resins. Resins with higher M_n_ values, such as FPI-3 (M_n_ = 2.87 × 10^5^ g/mol) and FPI-5 (M_n_ = 1.02 × 10^5^ g/mol), provided fine fibers with obviously higher d_av_ values. For low-M_n_ resins, such as FPI-1 (M_n_ = 6.45 × 10^4^ g/mol), thin fibers with lower d_av_ values were obtained. Meanwhile, spindle-shaped bulges were observed in the derived fibers. In view of the effects of the fiber diameters on the optical properties of UFMs, the currently developed FPI UFMs consisted of the fibers with thin diameters might exhibit high whiteness and optical reflectance.

The water contact angles (WCA) of FPI UFMs were measured and the results are shown in Table 2 and Figure S4 (Supplementary Materials). Generally, the electrospun fabrics usually exhibited somewhat high humidity absorptions due to the porous nature of the materials. Thus, various methodologies were performed to increase the hydrophobicity of electrospun UFMs, either by structural modification [34] or by blending with inorganic nanoparticles [35]. It can be seen from Figure S4 (Supplementary Materials) that all FPI UFMs exhibited a good hydrophobic nature with the increasing WCA values of FPI-4 < FPI-3 < FPI-5 < FPI-2 < FPI-1. The highest WCA value for FPI-1 might be due to the highest fluoro contents in the polymer. For FPI-5, besides the effects of the fluoro-containing units in the dianhydride moiety, the low-polar *tert*-butyl-substituted cyclohexyl units in the diamine moiety also contributed to the high hydrophobicity of the polymer. The hydrophobic or low moisture-sensitive nature of the current UFMs is beneficial for their practical applications in optoelectronic devices.

### 2.2. Optical Properties

**Figure 6:**
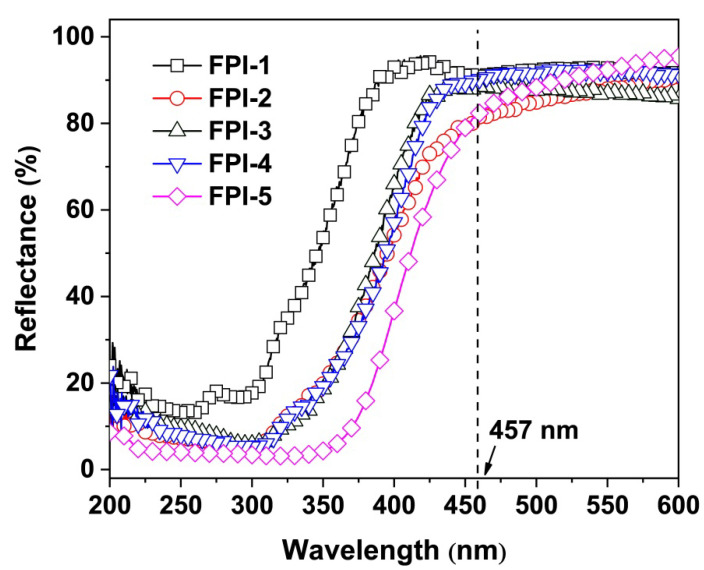
UV-Vis reflective spectra of FPI UFMs.

The optical properties of FPI UFMs, mainly the optical reflectance and CIE Lab color parameters, were investigated and the results are shown in Table 2. Figure 6 exhibits the optical reflectance plots of the FPI UFMs, in which the reflectance values at the wavelength of 457 nm (R_457_) were recorded. FPI UFMs showed good optical reflectance with the R_457_ values higher than 80%, which were obviously higher than that of the PI-ref (R_457_ = 37.3%). FPI UFMs exhibited R_457_ values with the increasing order of FPI-5 (80.5%) < FPI-2 (81.7%) < FPI-3 (88.1%) < FPI-4 (90.5%)<FPI-1 (91.8%). As mentioned before, the optical reflectance features of electrospun UFMs are simultaneously affected by the surface physical micromorphology and the chemical compositions of the polymers. As for the effects of the chemical structures, it is known that the lower CT interactions in the molecular chains of the polymers usually result in the lower absorptions of the polymers to the visible light. The CT interactions could be roughly estimated by the energy level gap (E_g_) between the lowest unoccupied molecular orbital (LUMO) energy levels (ε_LUMO_) and the ε_HOMO_ for the repeating units of the PIs (E_g_ = ε_LUMO_ − ε_HOMO_) [36]. The lower the E_g_ values, the more significant the CT interactions in the polymers, and the more significant the absorption of visible light by the polymers. The E_g_ values of the FPIs showed the increasing order of FPI-5 < FPI-2 < FPI-4 < FPI-3 < FPI-1 according to the simulation results. This trend is basically consistent with the R_457_ values of FPI UFMs. FPI-1, with the highest fluoro contents, showed the highest E_g_ and R_457_ values, indicating the efficiently prohibited CT interactions by the electronegative –CF_3_ groups, both in the dianhydride and the diamine moieties. Contrarily, FPI-5 with the lowest content of –CF_3_ group showed the lowest E_g_ and R_457_ values.

**Figure 7:**
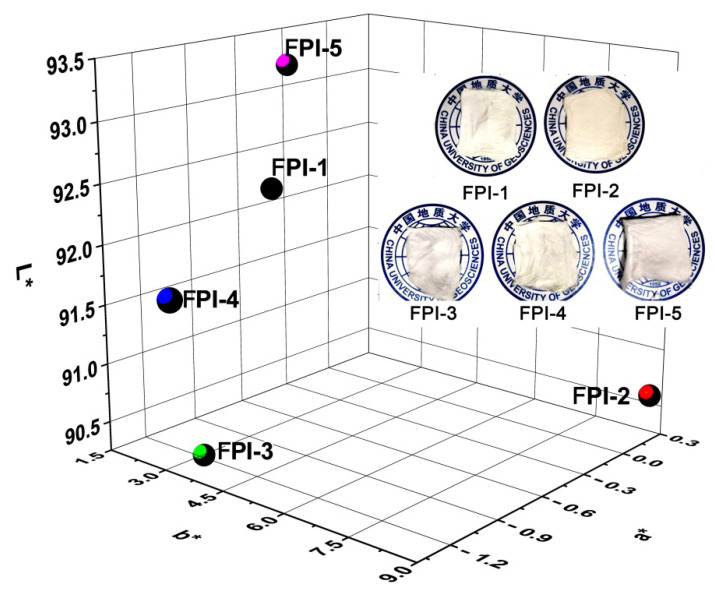
3D CIE Lab color parameters of FPI UFMs (Insert: Appearance of FPI UFMs).

The CIE Lab color parameters of FPI UFMs were further detected and the three-dimensional plots are shown in Figure 7, together with the appearance of UFMs. The whiteness indices (WI) of FPI UFMs were calculated from the L^*^, a^*^, and b^*^ parameters of FPI UFMs and are listed in Table 2. All FPI UFMs showed WI values higher than 80.00, which were apparently higher than that of the PI-ref (WI = 59.02). This was mainly attributed to the low CT interactions in the fluoro-contaning polymers. In addition, FPI UFMs exhibited decreasing WI values with the order of FPI-5 > FPI-1 > FPI-4 > FPI-3 > FPI-2. Interestingly, FPI-5 with the lowest optical reflectance at the wavelength of 457 nm, and the highest CT interactions, exhibited the highest WI value. This indicates that the incorporation of the non-conjugated tert-butyl-substituted cyclohexyl structure in FPI-5 efficiently reduced the absorption of the polymer in the visible light region, although it showed higher absorption in the wavelength range close to the ultraviolet region. Actually, it can be deduced from Figure 6 that, in the high-wavelength region, such as the wavelegnth of 600 nm, FPI-5 UFM showed the highest optical reflectance.

### 2.3. Thermal Properties

**Figure 8:**
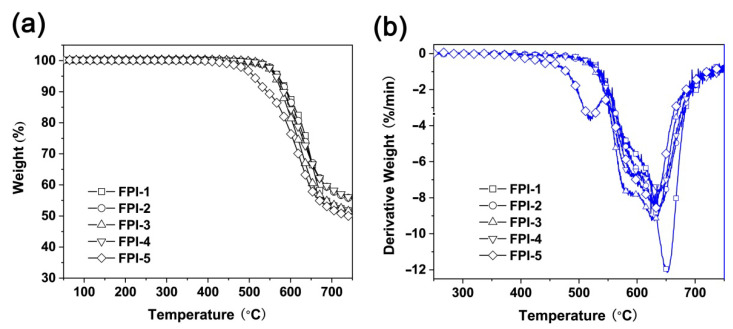
Thermal decomposition behaviors of FPI UFMs. (**a**) TGA and (**b**) DTG.

The thermal stability of the FPI UFMs was evaluated by TGA and DSC measurements, and the thermal data are summarized in Table 2. According to the TGA and derivative TGA (DTG) curves of the FPI UFMs shown in Figure 8, all UFMs showed good thermal stability before 400 °C in nitrogen. FPI UFMs exhibited a 5% weight loss temperatures (T_5%_) in the range of 510.5–569.6 °C and residual weight ratios higher than 50 wt% at 700 °C. All FPI UFMs showed the single-stage thermal decomposition behaviours, except that of FPI-5. For FPI-1–FPI-4 UFMs, the maximum thermal decomposition occurred in the temperature range of 630–650 °C. However, for FPI-5, a two-stage thermal decomposition was observed. The first was detected around 520 °C, which was mainly due to the thermal decomposition of the side chains. The second was found at 635 °C, which was ascribed to the main chain decomposition in the polymer.

**Figure 9:**
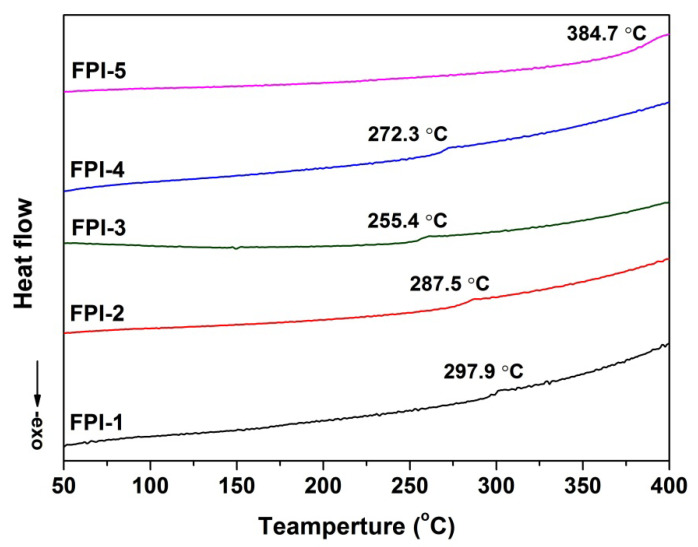
DSC plots of FPI UFMs.

The glass transition temperatures (T_g_) of the FPI UFMs were measured by DSC, and the plots are shown in Figure 9. For FPI-1–FPI-4, incorporation of the bulky hexafluoroisopropyl in the dianhydride and trifluoromethyl groups in the diamines endowed the polymers with high T_g_ values in the range of 255.4–297.9 °C. FPI-2–FPI-4 UFMs exhibited inferior T_g_ values compared to FPI-1 due to the flexible ether linkages in the molecular structures. Interestingly, FPI-5 did not show a clear glass transition during the measurement until 384.7 °C, at which point an unconspicuous heat-flow change was detected. It has been reported that the FPI derived from 6FDA and *meta*-phenylene diamine (mPDA) showed a T_g_ value of 301 °C [37]. Incorporation of the *tert*-butyl-substituted cyclohexyl structure via rigid ester linkage might increase the T_g_ of the polymer.

## 3. Materials and Methods

### 3.1. Materials

The aromatic dianhydride, 4,4′-(hexafluoroisopropylidene)diphthalic anhydride (6FDA) with a polymerization-grade purity (≥99.5%) was purchased from ChinaTech (Tianjin) Chem. Co. Ltd. (Tianjin, China), and dried at 180 °C in vacuo for 24 h prior to use. The fluoro-containing diamines, including 3,5-diaminobenzotrifluoride (TFMDA), 2′-trifluoromethyl-3,4′-oxydianiline (3FODA), 1,4-bis[(4-amino-2-trifluoro- methyl)phenoxy]benzene (6FAPB), and 4,4′-bis[(4-amino-2-trifluoromethyl)phenoxy]- biphenyl (6FBAB) were purchased from Changzhou Sunlight Pharmaceutical Co., Ltd. (Changzhou, Jiangsu, China) and used directly. Electronic grade *N*-methyl-2-pyrrolidinone (NMP) and *N,N*-dimethylacetamide (DMAc) were purchased from Greenda Chem. Co. Ltd., China (Hangzhou, Zhejiang, China) and used as received. The other reagents and materials were purchased from Sinopharm Chem. Reagent Co. Ltd. (Shanghai, China) and used as received.

### 3.2. Measurements

Inherent viscosity was measured using an Ubbelohde viscometer (Pingxuan Scientific Instrument Co. Ltd., Shanghai, China) with a 0.5 g/dL NMP solution at 25 °C. Attenuated total reflectance-Fourier transform infrared (ATR-FTIR) spectrum was obtained on an Iraffinity-1S FT-IR spectrometer (Shimadzu, Kyoto, Japan). ^1^H-NMR was performed on an AV 400 spectrometer (Tokyo, Japan), operating at 300 MHz and using DMSO-d_6_ as the solvent and tetramethylsilane as the reference. Ultraviolet-visible (UV-Vis) spectra of the PI UFMs were recorded on a Hitachi U-3210 spectrophotometer (Tokyo, Japan) at room temperature with a reflectance mode. The number average molecular weight (M_n_) and weight average molecular weight (M_w_) of the PI resins were measured using a gel permeation chromatography (GPC) system (Shimadzu, Kyoto, Japan) with a Shodex KF-804 column (Tokyo, Japan). HPLC grade NMP was used as the mobile phase at a flow rate of 1.0 mL/min. Polystyrene (Shodex, Type: SM-105, Showa Denko Co. Ltd., Tokyo, Japan) was used as the standard. Wide-angle X-ray diffraction (XRD) was conducted on a D/max-2500 X-ray diffractometer (Rigaku, Tokyo, Japan). The micro-morphologies of the PI UFMs were detected via a JSM-6700F (JEOL, Tokyo, Japan) field emission scanning electron microscopy (FE-SEM). The color parameters of the PI UFMs were measured using an X-rite color i7 spectrophotometer (Grand Rapids, MI, USA) and calculated according to a CIE (Comission Internationale del’Eclairage) Lab equation. L^*^ is the lightness, where 100 means white and 0 implies black. A positive a^*^ means a red color, and a negative one indicates a green color. A positive b^*^ means a yellow color, and a negative one indicates a blue color. The whiteness indices (WI) of the PI UFMs were calculated as follows: WI = 100 − [(100 − L^*^)^2^ + a^*2^ + b^*2^]^1/2^, where WI standards for whiteness index, L^*^ standards for lightness, a^*^ and b^*^ stand for chromaticity coefficient. The water contact angles (WCAs) of the PI UFMs were measured using the sessile drop method using an OCA-40Micro (Stuttgart, Germany) contact angle analyzer. Five samples were measured and the average WCA values were then obtained. A drop of 5 μL deionized water was used for the measurements. Thermogravimetric analysis (TGA) was performed on a TA-Q50 thermal analysis system (New Castle, DE, USA) at a heating rate of 20 °C min^−1^ in nitrogen. Differential scanning calorimetry (DSC) was carried on a TA-Q 100 thermal analysis system (New Castle, DE, USA) at a heating rate of 10 °C min^−1^ in nitrogen.

Solubility of the PI resins in various solvents was determined by adding 1.0 g of the PI resin into 9.0 g of the tested solvent at room temperature (10 wt% solid content). The mixture was mechanically stirred at 25 °C for 24 h. The solubility was determined visually as three grades: completely soluble (++), partially soluble (+), and insoluble (-). The complete solubility is defined as a homogenous and clean solution is obtained, in which no phase separation, precipitation or gel formation is detected.

### 3.3. Synthesis of 4′-tert-butyl-cyclohexyl-3,5-diaminobenzoate (DABC)

DABC was synthesized in our laboratory with 3,5-dinitrobenzyl chloride and 4′-*tert*-butylcyclohexyol as the starting materials, according to the similar procedure reported in the literature, and purified by recrystallization from absolute ethanol [38]. DABC was obtained as white needles with a purity of 99.5 % according to the gas chromatography analysis. Yield: 57%. Melting point: 192.0 °C (DSC peak temperature). FT-IR (KBr, cm^−1^): 3472, 3441, 3341, 3210, 2951, 2862, 1694, 1597, 1458, 1366, 1254, 1196, and 775. ^1^H-NMR (DMSO-*d*_6_, ppm): 6.41–6.34 (m, 2H), 6.01–5.97 (m, 1H), 4.95–4.89 (m, 4H), 4.69–4.61 (m, 1H), 2.04–1.96 (m, 2H), 1.81–1.73 (m, 2H), 1.36–1.30 (m, 2H), 1.14–0.98 (m, 3H), and 0.85–0.79 (m, 9H). ^13^C-NMR (DMSO-*d*_6_, ppm): 166.7, 149.7, 131.7, 104.1, 73.5, 46.9, 32.5, 32.3, 27.9, and 25.6. Elemental analysis: calculated for C_17_H_26_N_2_O_2_ (M_w_: 290.40 g/mol): C, 70.31%, H, 9.02%, N, 9.65%; Found: C, 70.37%, H, 9.05%, N, 9.55%.

### 3.4. Preparation of PI Resins and Electrospun UFMs

PI resins were prepared from 6FDA and the diamines via a two-step chemical imidization procedure, which could be illustrated by the synthesis of FPI-5 (6FDA-DABC). DABC (14.5200 g, 50 mmol) and DMAc (100.0 g) were filled into a three-necked 500 mL flask equipped with a mechanical stirrer, a nitrogen inlet, and a cold-water bath. The diamine solution was stirred at 10–15 °C for 30 min under nitrogen and 6FDA (22.2120 g, 50 mmol) was added in one batch together with an additional DMAc (10.2 g). The solid content of the polymerization mixture was controlled to be 25 wt%. The cold bath was removed after 4 h and the viscous solution was further stirred at room temperature for another 20 h. A pale-yellow poly(amic acid) (PAA) solution was obtained. Then, acetic anhydride (30.6 g, 300 mmol) and pyridine (19.0 g, 240 mmol) were added into the PAA solution with vigorous stirring. The chemical imidization procedure was performed at room temperature for another 24 h. At last, the polymerization mixture was slowly poured into an excess of aqueous ethanol solution (75 wt%). FPI-5 resin was then precipitated from the PI solution. The resin was thoroughly immersed into the ethanol solution and then collected. The wet resin was dried under vacuum at 120 °C for 24 h to afford the FPI-5 resin as a pale-yellow fibrous resin. Yield: 34.06 g (97.5%).

The other PI resins, including FPI-1 (6FDA-TFMDA), FPI-2 (6FDA-3FODA), FPI-3 (6FDA-6FAPB), and FPI-4 (6FDA-6FBAB) were prepared according to a similar procedure as mentioned above, except DABC was replaced by TFMDA for FPI-1, by 3FODA for FPI-2, by 6FAPB for FPI-3, and by 6FBAB for FPI-4, respectively.

### 3.5. Electrospinning Fabrication

The FPI UFMs were fabricated via a one-step electrospinning procedure reported in the literature [39]. FPI-5 UFM was used as an example to present the detailed procedure. Fully dried FPI-5 resin was dissolved into ultra-dry DMAc at a solid content of 15 wt%. The obtained FPI-5 solution was filtered through a 0.45 μm Teflon filtration membrane to remove any undissolved impurities. Then, the FPI-5 solution was filled into a 5 mL high-density polyethylene syringe equipped with a spinneret with an inner diameter of 0.21 mm. A syringe pump was used to squeeze out the PI solution through the needle spinneret at a speed of 0.2 mL/h. A voltage of 15 kV was applied between the syringe and the collector. The distance between the spinneret and the grounded plate collector was 15 cm. The collector was the aluminum foil covered onto the rotating drum (diameter: 10 cm; length: 30 cm; speed: 200 rpm). The relative humidity in the electrospinning apparatus was controlled to be 35 ± 5%. The FPI-5 fibers were continuously deposited onto the aluminum foil and formed the final FPI-5 UFM. After the electrospinning fabrication, the FPI-5 NFM was placed in a circulating oven at 200 °C for 1 h to remove the residual solvent.

The other FPI UFMs, including FPI-1 (6FDA-TFMDA), FPI-2 (6FDA-3FODA), FPI-3 (6FDA-6FAPB), and FPI-4 (6FDA-6FBAB), were prepared according to a similar procedure. The PI-ref UFM was made by a two-step procedure according to the literature [25]. PAA UFM was first prepared and then thermally dehydrated at 300 °C to afford the PI-ref UFM.

## 4. Conclusions

Fluoro-containing electrospun PI UFMs with desired features for advanced optoelectronic applications were designed and prepared in the current work. For example, in one of research interests, flexible or foldable UFMs with heat-induced fluorescent features at elevated temperatures for industrial labelling or accurate identification are required. In such applications, the properties of high whiteness, good thermal stability, and low moisture sensitivity are required for the UFMs. The currently developed electrospun FPI UFMs possessed the basic requirements for functional UFMs, including WI values higher than 87.00, T_g_ over 255 °C, and WCA values higher than 105°. Thus, FPI UFMs might be good candidates in the applications mentioned above, and the detailed fabrication of the devices with the FPI UFMs was performed in our laboratory.

## Figures and Tables

**Table 1:** Inherent viscosities, molecular weights, and solubility of FPI resins.

PI	[η]_inh_^a^ (dL/g)	Molecular Weight			Solubility				
		M_n_ (×10^4^ g/mol)	M_w_ (×10^4^ g/mol)	PDI	NMP	DMAc	GBL	CPA	CHCl_3_
FPI-1	0.84	5.37	10.56	1.65	++	++	++	++	++
FPI-2	0.97	6.45	11.63	1.80	++	++	++	++	++
FPI-3	1.13	10.12	20.48	2.02	++	++	++	++	+
FPI-4	1.16	10.21	17.70	1.73	++	++	++	++	+
FPI-5	1.22	28.70	47.47	1.65	++	++	++	+	+

^a^ Inherent viscosities measured with a 0.5 g dL^−1^ PI solution in NMP at 25 °C; M_n_: number average molecular weight; M_w_: weight average molecular weight; PDI: polydispersity index, PDI = M_w_ / M_n_; ++: Soluble; +: partially soluble; GBL: γ-butyrolactone; CPA: cyclopentanone.

**Table 2:** Optical and thermal properties of FPI UFMs.

PI	d_av_ (nm)	WCA (°)	Optical Properties					Thermal Properties			
			R_457_ (%)	L^*^	A^*^	B^*^	WI	T_5%_ (°C)	T_max_ (°C)	R_w700_ (%)	T_g_ (°C)
FPI-1	360	134.5 ± 2.1	91.8	92.28	−0.65	2.64	91.82	569.6	651.8	53.6	297.9
FPI-2	665	130.5 ± 1.3	81.7	90.64	0.21	8.96	87.04	569.1	637.0	58.2	287.5
FPI-3	1937	117.5 ± 2.2	88.1	90.16	−1.10	2.65	89.75	562.2	629.7	53.9	255.4
FPI-4	1037	105.5 ± 1.7	90.5	92.01	−1.76	4.99	90.42	565.7	639.2	58.5	272.3
FPI-5	1363	119.8 ± 1.8	80.5	93.25	−0.44	2.21	92.88	510.5	519.5, 635.0	52.1	384.7
PI-ref	993	86.2 ± 2.6	37.3	84.65	1.62	37.96	59.02	ND	ND	ND	ND

d_av_: average fiber diameter; WCA: water contact angle; R_457_: Reflectance at the wavelength of 457 nm; L^*^, A^*^, B^*^: CIE color parameters; WI: whiteness; T_5%_: Temperatures at 5% weight loss; T_max_: Temperatures at which the highest decomposing ratio occurred; R_w700_: Residual weight ratio at 700 °C in nitrogen; T_g_: Glass transition temperatures. PI-ref: poly(pyromellitic dianhydride-oxydianiline) (PMDA-ODA); ND: Not detected.

## Data Availability

Data are contained within the article.

## Supplementary Materials

The following supporting information can be downloaded at: https://www.mdpi.com/article/10.3390/molecules27175447/s1, Figure S1: Optimized structures and the calculated HOMO energy levels (εHOMO) for the diamines; Figure S2: XRD patterns of FPI resins; Figure S3: SEM images of the FPI UFMs together with the average fiber diameters (*d*_av_). (**a**) FPI-1, (**b**) FPI-2, (**c**) FPI-3, (**d**) FPI-4, (**e**) FPI-5; Figure S4: Water contact angles of FPI UFMs. (**a**) FPI-1, (**b**) FPI-2, (**c**) FPI-3, (**d**) FPI-4, (**e**) FPI-5.

## Sample Availability

Samples of the compounds are available from the authors.
