# Supplementary material for: Molecular Design, Preparation, and Characterization of Fluoro-Containing Polyimide Ultrafine Fibrous Membranes with High Whiteness, High Thermal Stability, and Good Hydrophobicity
Source: Molecules. 2022 Aug 25;27(17):5447. doi: 10.3390/molecules27175447 (PMC9457758; doi:10.3390/molecules27175447)
Supplement: Supplementary file 1 [file molecules-27-05447-s001.zip › molecules-1875694-supplementary.pdf]

molecules

Supplementary Materials

Molecular Design, Preparation, and Characterization of Fluoro-Containing Polyimide Ultrafine Fibrous Membranes with High Whiteness, High Thermal Stability, and Good Hydrophobicity

Zhen Pan [1,†], Han-li Wang [2,†], Hao-ran Qi [1], Yan-shuang Gao [1], Xiao-lei Wang [1], Xin-xin Zhi [1], Yan Zhang [1], Xi Ren [1] and Jin-gang Liu [1,*]

[1] School of Materials Science and Technology, China University of Geosciences, Beijing 100083, China
[2] Shandong Huaxia Shenzhou New Material Co. Ltd., Shandong 256401, China
* Correspondence: liujg@cugb.edu.cn; Tel.: +86-10-8232-2972
† These authors contributed equally to this work.

Citation: Pan, Z.; Wang, H.-l.; Qi, H.-r.; Gao, Y.-s.; Wang, X.-l.; Zhi, X.-x.; Zhang, Y.; Ren, X.; Liu, J.-g. Molecular Design, Preparation, and Characterization of Fluoro-Containing Polyimide Ultrafine Fibrous Membranes with High Whiteness, High Thermal Stability, and Good Hydrophobicity. *Molecules* **2022**, *27*, 5447. https://doi.org/10.3390/molecules27175447

Academic Editor: Matthias Schnabelrauch

Received: 4 August 2022
Accepted: 23 August 2022
Published: 25 August 2022

Publisher's Note: MDPI stays neutral with regard to jurisdictional claims in published maps and institutional affiliations.

Copyright: © 2022 by the authors. Submitted for possible open access publication under the terms and conditions of the Creative Commons Attribution (CC BY) license (https://creativecommons.org/licenses/by/4.0/).

Supplementary Files:

Captions:

Figure S1. Optimized structures and the calculated HOMO energy levels (εHOMO) for the diamines.
Figure S2. XRD patterns of FPI resins.
Figure S3. SEM images of the FPI UFMs together with the average fiber diameters (d_{av}). (**a**) FPI-1, (**b**) FPI-2, (**c**) FPI-3, (**d**) FPI-4, (**e**) FPI-5.
Figure S4. Water contact angles of FPI UFMs. (**a**) FPI-1, (**b**) FPI-2, (**c**) FPI-3, (**d**) FPI-4, (**e**) FPI-5.

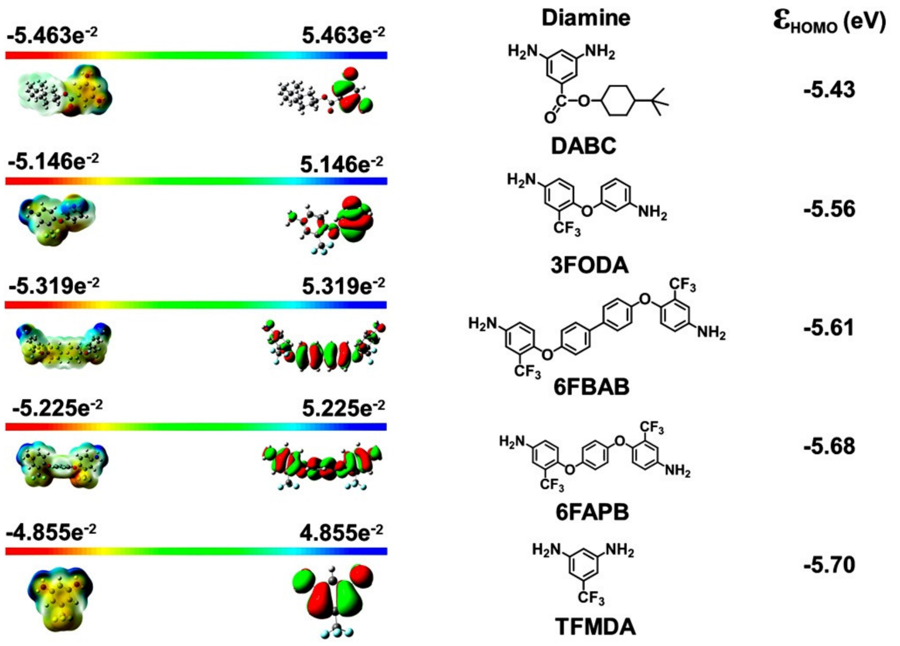

Figure S1. Optimized structures and the calculated HOMO energy levels (εHOMO) for the diamines.

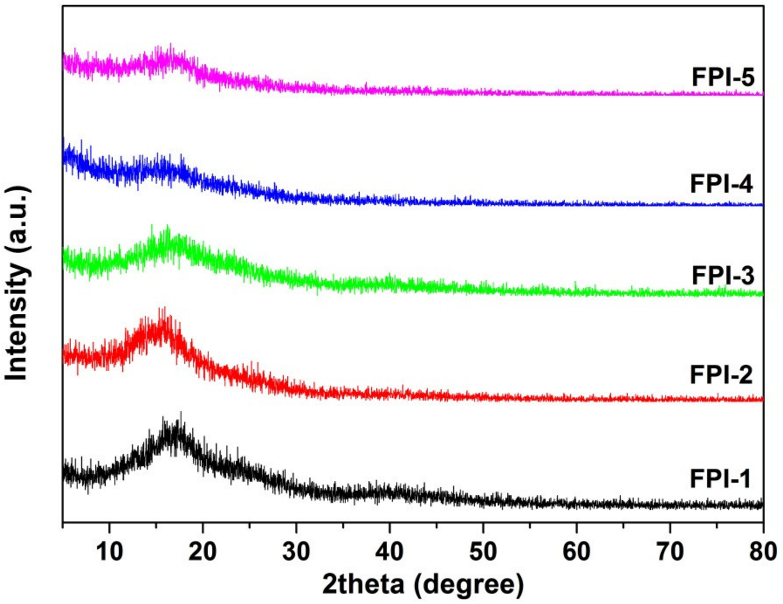

Figure S2. XRD patterns of FPI resins.

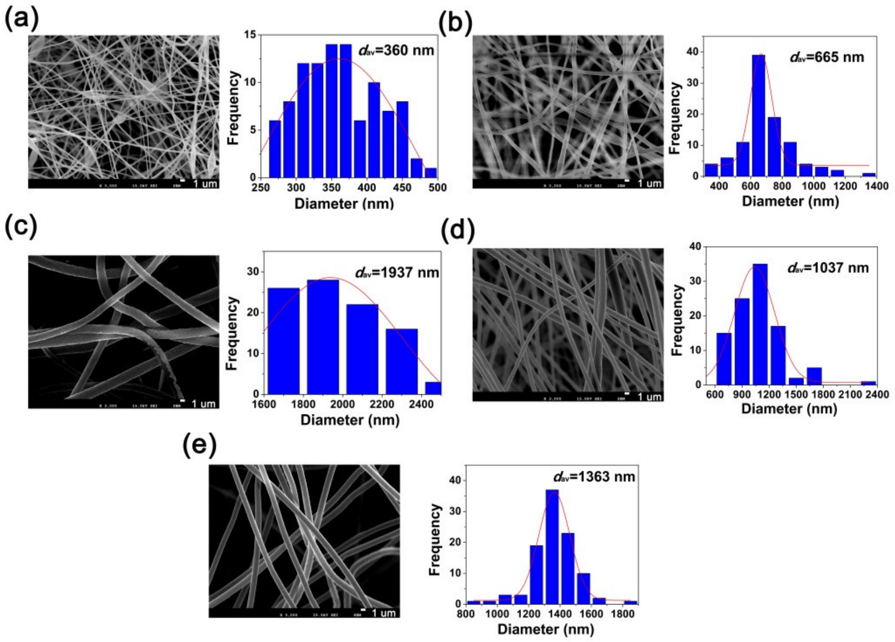

Figure S3. SEM images of the FPI UFMs together with the average fiber diameters (d_{av}). (**a**) FPI-1, (**b**) FPI-2, (**c**) FPI-3, (**d**) FPI-4, (**e**) FPI-5.

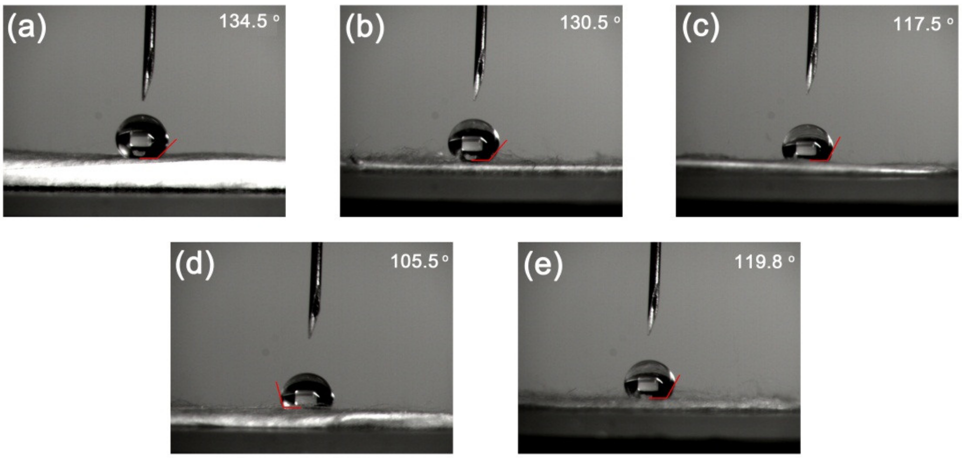

Figure S4. Water contact angles of FPI UFMs. (**a**) FPI-1, (**b**) FPI-2, (**c**) FPI-3, (**d**) FPI-4, (**e**) FPI-5.
